# All-suture anchor size and drill angle influence load to failure in a porcine model of subpectoral biceps tenodesis, a biomechanical study

**DOI:** 10.1186/s12891-024-07503-0

**Published:** 2024-05-23

**Authors:** Prisca S. Alt, Christian Marx, Sepp Braun

**Affiliations:** 1 Department of Handsurgery, University Hospital Basel, Basel, Switzerland; 2 Gelenkpunkt - Sports and Joint Surgery, Innsbruck, Austria; 3 Research Unit for Orthopedic Sports Medicine and Injury Prevention, Medical Informatics and Technology, UMIT - Private University for Health Sciences, Hall in Tirol, 6060 Austria

**Keywords:** Arthroscopy, Shoulder, SLAP, Tenotomy

## Abstract

**Background:**

Tenodesis of the long head of the biceps tendon is frequently performed in shoulder surgery, and all-suture anchors have become more popular as fixation methods. However, uncertainty still exists regarding the ultimate load to failure of all-suture anchors and the best insertion angle at a cortical humeral insertion point.

**Purpose:**

The purpose of this study was to compare the biomechanical characteristics of three types of all-suture anchors frequently used for biceps tenodesis. In addition, the influence of two different insertion angles was observed in a porcine humeri model.

**Methods:**

The ultimate load to failure and failure mode of three types of all-suture anchors (1.6 FiberTak®, 1.9 FiberTak®, 2.6 FiberTak®, Arthrex®) applicable for subpectoral biceps tenodesis were evaluated at 90° and 45° insertion angles in 12 fresh-frozen porcine humeri. The anchors were inserted equally alternated in a randomized manner at three different insertion sites along the bicipital groove, and the suture tapes were knotted around a rod for pullout testing. In total, 36 anchors were evaluated in a universal testing machine (Zwick & Roell).

**Results:**

The 2.6 FiberTak® shows higher ultimate loads to failure with a 90° insertion angle (944.0 *N* ± 169.7 N; 537.0 *N* ± 308.8 N) compared to the 1.9 FiberTak® (677.8 *N* ± 57.7 N; 426.3 *N* ± 167.0 N, p-value: 0.0080) and 1.6 FiberTak® (733.0 *N* ± 67.6 N; 450.0 *N* ± 155.8 N, p-value: 0.0018). All anchor types show significantly higher ultimate loads to failure and smaller standard deviations at the 90° insertion angle than at the 45° insertion angle. The major failure mode was anchor pullout. Only the 2.6 FiberTak® anchors showed suture breakage as the major failure mode when placed with a 90° insertion angle.

**Conclusions:**

All three all-suture anchors are suitable fixation methods for subpectoral biceps tenodesis. Regarding our data, we recommend 90° as the optimum insertion angle.

**Clinical relevance:**

The influence of anchor size and insertion angle of an all-suture anchor should be known by the surgeon for optimizing ultimate loads to failure and for achieving a secure fixation.

## Background

Suprapectoral and subpectoral biceps tenodesis are the two most frequently used techniques to address anterior shoulder pain or biceps tendon injuries [1]. However, subpectoral tenodesis is reported to be more effective in long-lasting pain reduction and fewer revision rates [1]. As a fixation method, all-suture anchors (ASAs) have become more popular in recent years. Multiple studies have confirmed similar or slightly lower pullout strength for ASAs compared to conventional suture anchors (CSA) [2–8], button systems [8–10] or interference screws [8, 11–13]. In contrast to other fixation implants, ASAs preserve bone stock, reduce soft-tissue damage and torsional forces due to smaller drilling holes and therefore minimize fracture risk [9, 14, 15] and improve postoperative imaging [16–18]. There are still remaining risks for failure with the suture-tendon interface as a critical side [8–10]. The most common failure mode is tendon tearing [9, 10, 13]. Studies regarding the ultimate load to failure of different types of ASAs in varying insertion angles for subpectoral biceps tenodesis are currently lacking. Thus far, all published data regarding the optimal insertion angle for ASAs focus on RC (rotator cuff) repair and propose an optimal insertion angle of 90° to the bone surface [2, 19]. However, when performing minimally invasive subpectoral biceps tenodesis, the surgeon can be blocked by the pectoralis major when aiming for a perpendicular insertion of the anchor and therefore might rather aim for a 45° insertion angle. Since the cortical thickness and the tendon traction angle play major roles in ASA fixation [18, 20], we expect different biomechanical properties in fixation strength in the bicipital groove with an approximately 3 mm cortex compared to the greater tuberosity with partially only 0.3 mm cortex [21].

The purpose of this study was to compare the biomechanical characteristics of three available sizes of ASAs (1.6 FiberTak®, 1.9 FiberTak®, 2.6 FiberTak®, Arthrex GmbH, Munich, Germany) used for biceps tenodesis and the influence of two different insertion angles in a porcine humeri model. We hypothesize that all anchor types show similar pullout strength with a decreased ultimate load to failure at a 45° insertion angle compared to a 90° insertion angle.

## Methods

A total of 12 paired porcine cadaveric humeri were used in this study and randomly divided into two groups. All specimens were six months of age and obtained from a local butcher. The humeri were carefully dissected of soft tissue, stored in a plastic bag, and kept in a freezer at a constant temperature of -20 °C. One day prior to biomechanical testing, they were thawed at room temperature for 24 h. All humeri were transected at the mid-diaphysis level with a band saw to enable suture traction along the physiological pull direction of the biceps tendon. The humeri were then potted in a specially designed jig and fixed by a slowly hardening epoxy resin.

### All-suture anchors

**Fig. 1:**
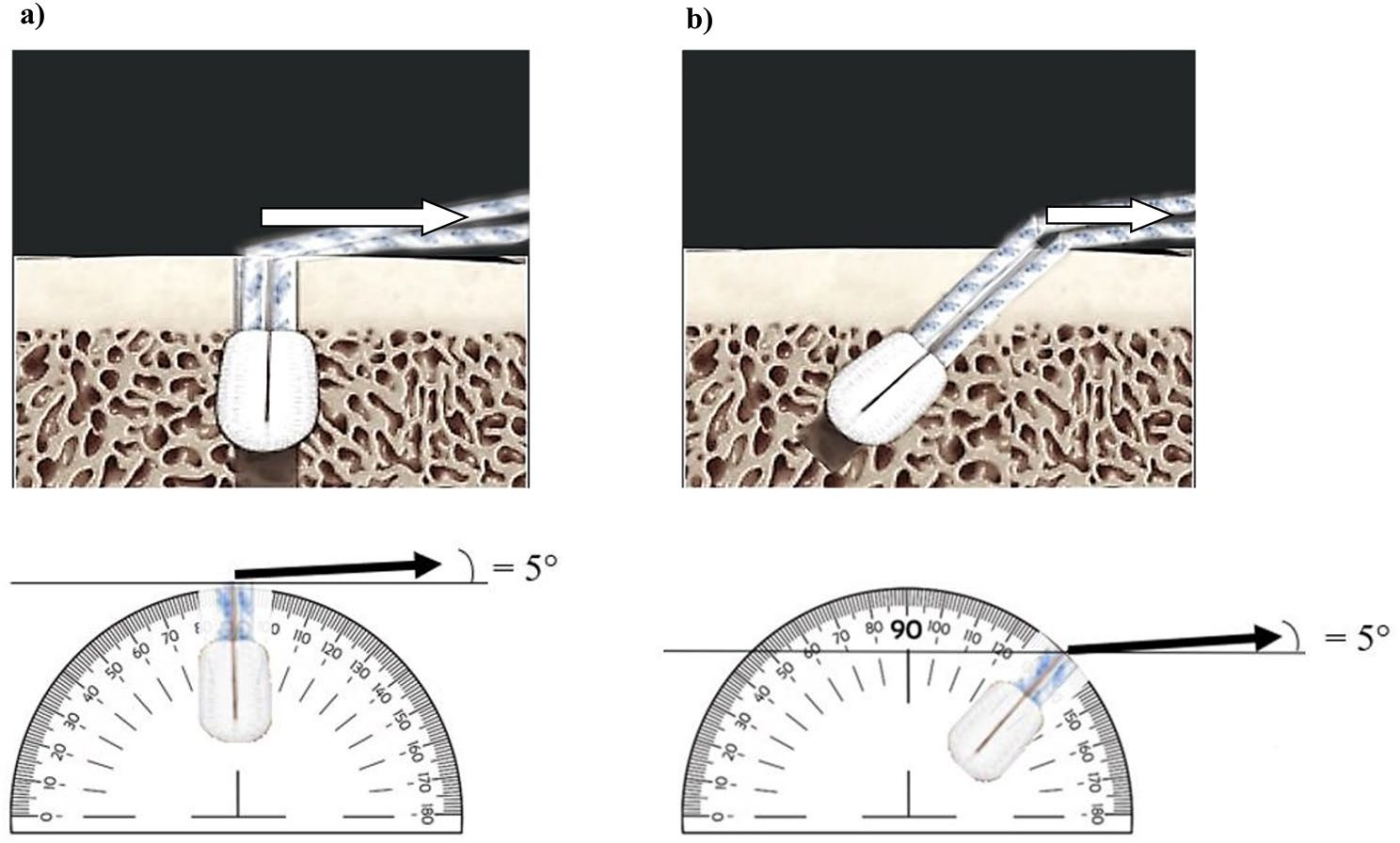
Insertion and traction angle (**a**) 90° Insertion angle and traction along the physiological biceps contraction direction = 5° (**b**) 45° Insertion angle and traction along the physiological biceps contraction direction = 5°

Three types of ASAs from the manufacturer Arthrex® were selected for testing: 1.6 mm FiberTak Suture Anchor®, 1.9 mm FiberTak Biceps Implant System® and 2.6 mm FiberTak Anchor DR®. All ASAs were double-loaded with 1.3 mm suture tapes, had the same deployment mechanism and formed a suture ball when tightened. The anchors were inserted into the porcine humeri following the manufacturer’s instructions as follows: A pilot hole was drilled with the associated drill through a custom-made 3D printed drill guide at 90° and 45° angles directed to the bone surface of the bicipital groove (Fig. 1). The anchors were inserted equally alternated in a randomized manner at three different insertion sites to minimize the influence of position and cortex characteristics at the insertion site. The insertion sites were placed at a 1.5 cm distance along the bicipital groove, which has been previously described to be a distance without biomechanical impact [22].

Suture strands were pulled for deployment, and anchor slippage occurred until the anchor was locked to the intramedullary cortex. In total, we tested 6 ASAs for each position and insertion angle in 12 porcine humeri (Fig. 1). Institutional review board approval was not needed for this study.

### Biomechanical testing

**Fig. 2:**
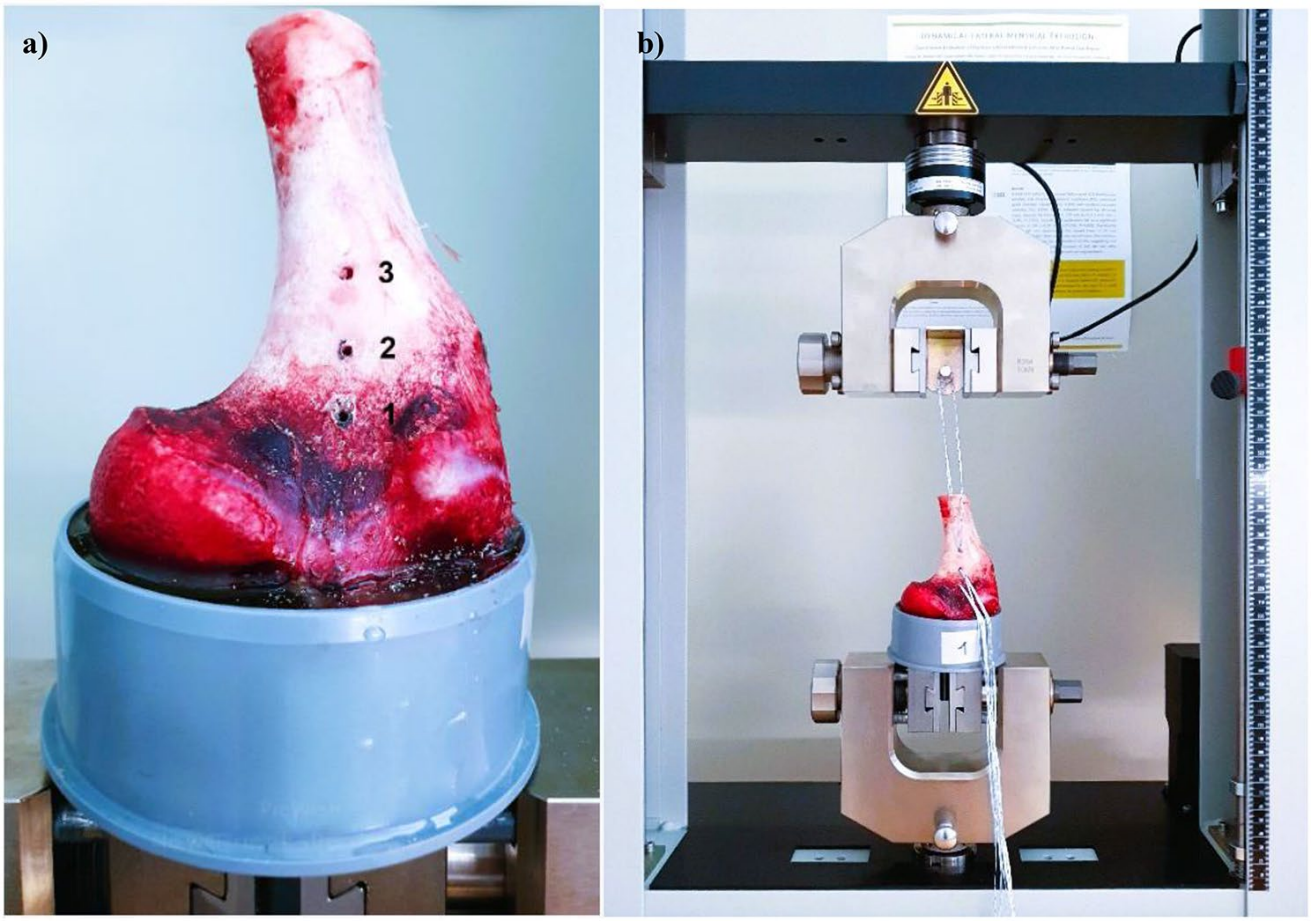
Anchor Positioning and Biomechanical Testing (**a**) Potted porcine humerus and alternated anchor positioning in the bicipital groove (**b**) Biomechanical setup with a universal testing machine (ZwickRoell type Z010)

Pullout tests were performed using a universal testing machine type Z010 (ZwickRoell GmbH & Co. KG, Ulm, Germany). Each strand of the suture tapes was winded thrice around a rod and hand tied with knots. The rod was attached to the load cell, with the interval between the ASA and rod standardized to 12 cm. Suture tapes were pulled transverse to the surface of the humerus, resembling the physiologic traction of the biceps muscle (Fig. 2). The anchor was preloaded to 100 N with an extension rate of 1 mm/s to ensure full engagement of the anchor to the bone. Then, the anchor was pulled at a crosshead speed of 1 mm/s according to previous studies [23, 24]. The load prior to sudden testing cessation or gradual load decrease caused by complete anchor pullout or suture tape breakage was recorded as the ultimate load to failure. The failure mode for each specimen was documented.

### Statistical analysis

MP® version 4.3 (SAS Institute Inc., Cary, NC, USA) was used for the statistical analysis of the results. The Shapiro-Wilk test was conducted to determine the normality of the distribution (Shapiro and Wilk, 1965), while Levene’s test was used to determine the homoscedasticity (Levene, 1960).

Three-way analysis of variance (ANOVA) was carried out to compare the Fmax of positions, number of anchors, insertion angle and the interaction between these factors. Since there were no interaction effects, a one-way ANOVA was performed for each factor. Means were compared with the Tukey post-hoc (HSD) test and Student’s t-test with *P* < 0.05 respectively.

## Results

**Fig. 3:**
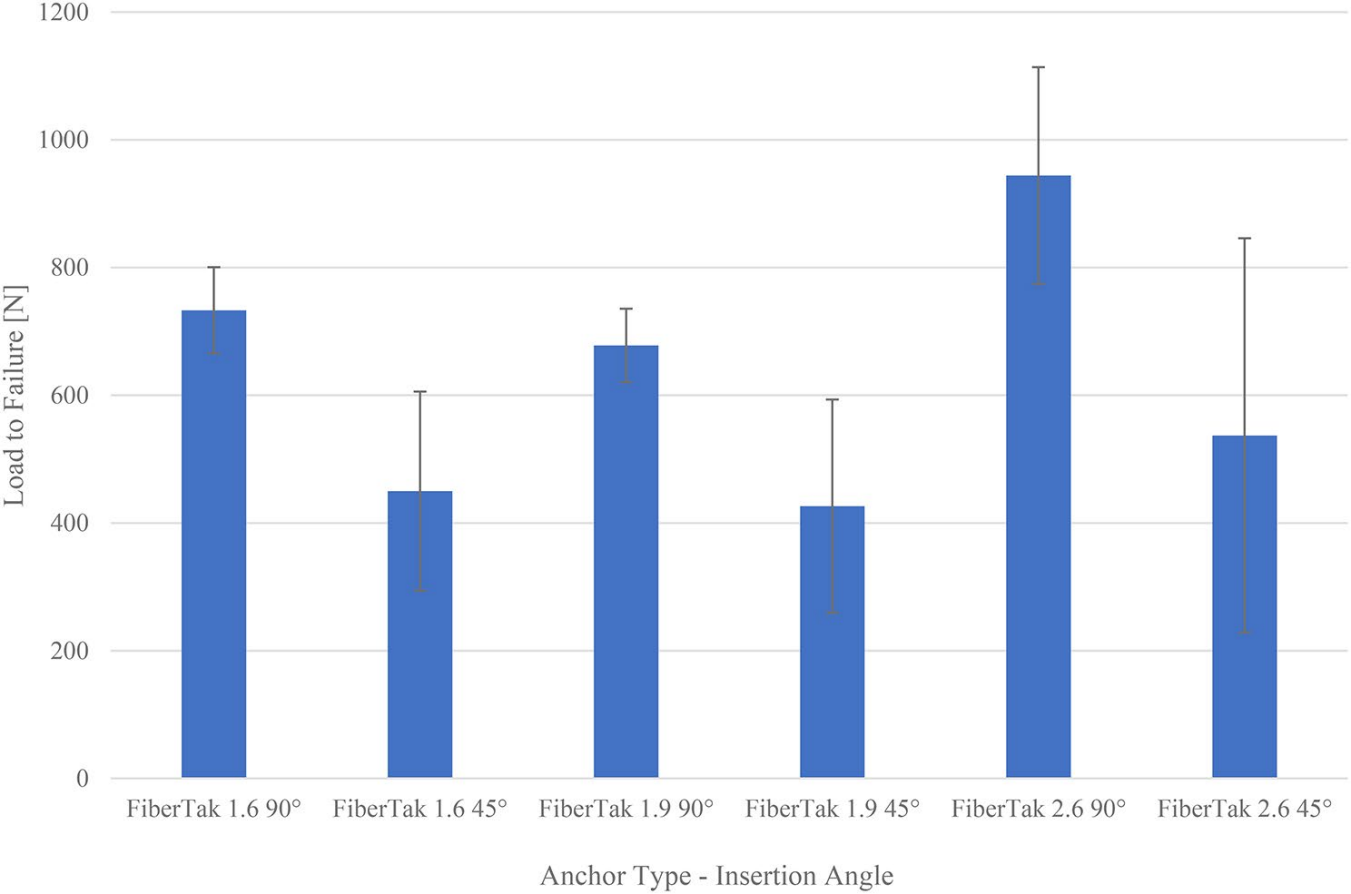
Ultimate load to failure according to the anchor type and insertion angle in N (Newton), presented as the mean ± standard deviation of the mean

A comparison of the three ASAs regardless of the insertion angle reveals a higher ultimate load to failure of the 2.6 FiberTak**®** than the 1.9 FiberTak**®** (p-value: 0,01384) but not the 1.6 FiberTak**®** (Table 1). The difference in the mean ultimate load to failure of the 2.6 FiberTak**®** compared to the 1.9 FiberTak**®** at 90° is significantly higher (p-value: 0.0080) than that of the 1.6 FiberTak**®** at 90° (p-value: 0.0018). Other individual comparisons between anchor types associated with the insertion angle revealed no further significant differences. However, all anchor types show significantly higher ultimate loads to failure and smaller standard deviations in the 90° insertion angle than in the 45° insertion angle (p-value: 0.0407, Table 1; Fig. 3).

**Table 1:** Descriptive data of ultimate load to failure according to the anchor type and insertion angle in Newtons (N)

	1.6 FiberTak® (*n* = 12)	1.9 FiberTak® (*n* = 12)	2.6 FiberTak® (*n* = 12)
90° Insertion	733.0 ± 67.6 (652–851)	677.8 ± 57.7 (591–753)	944.0 ± 169.7 (643–1174)
45° Insertion	450.0 ± 155.8 (245–615)	426.3 ± 167.0 (197–753)	537.0 ± 308.8 (145–947)

NOTE: Data are presented as the mean ± standard deviation of the mean

Two different failure modes were observed. All but one ASA of the 1.6 FiberTak® and 1.9 FiberTak® failed by anchor pullout at 90° and 45° insertion angles. The 2.6 FiberTak® failed in 5 out of 6 cases at a 90° insertion angle by suture breakage, whereas at a 45° insertion angle, the 2.6 FiberTak® failed only in 2 out of 6 cases by suture breakage and in 4 cases by anchor pullout (Table 2).

**Table 2:** Failure mode according to the anchor type and insertion angle

		1.6 FiberTak® (*n* = 12)	1.9 FiberTak® (*n* = 12)	2.6 FiberTak® (*n* = 12)
90° Insertion	Pullout Suture breakage	5/6 1/6	6/6 0/6	1/6 5/6
45° Insertion	Pullout Suture breakage	6/6 0/6	6/6 0/6	4/6 2/6

## Discussion

The main findings of this study were that [1] the 2.6 FiberTak® shows a significantly higher ultimate load to failure than the 1.9 FiberTak® (p-value: 0.0080) and 1.6 FiberTak® (p-value 0.0018) at a 90° insertion angle [2]. The ultimate load to failure decreases with a 45° insertion angle for all three anchor types (p-value: 0.0407), and the standard deviation increases [3]. The major failure mode is anchor pullout in all groups of the 1.9 FiberTak® and 1.6 FiberTak®, whereas suture breakage occurred in 5 out of 6 cases in 90° inserted 2.6 FiberTak®, but only twice in 45° inserted 2.6 FiberTak® as these failed by pull-out. All ASAs can be regarded as suitable fixation methods for subpectoral biceps tenodesis since all ultimate loads to failure exceed estimated necessary forces of 110 N for daily activities, which is the force to hold 1 kg in 90° of elbow flexion [10, 25]. Therefore, we recommend a 90° insertion angle to maximize pullout strength.

There are multiple possible influences on the ultimate load to failure of ASAs. Comparisons between studies are difficult since these vary in suture anchor design, bone material, bone density, insertion sites, traction angle, insertion angles and testing protocols as well as biomechanical interpretation. In our study, we found higher ultimate loads to failure in all tested anchors compared to prior biomechanical studies, which presented mean ultimate loads to failure for different types of ASAs ranging between 104 and 618 N [5, 18–20, 26–28]. Our results may exceed the results from preexisting studies due to the consistently stronger bone quality of young porcine specimens [5, 26, 29, 30], insertion site along the bicipital groove with thicker cortical bone [30], double-loaded ASAs [28] and traction in the physiologic direction of the biceps force vector instead of the anchor insertion direction [10, 19].

Additionally, other influences must be considered. In a biomechanical study, Noorazizi et al. revealed greater cortical destruction due to microfractures around angled drilling holes compared to perpendicular drilling holes [31], causing an overall greater drilling hole size. Euler et al. reported that eccentric placement of a drilling hole resulted in a 25% reduction in humeral shaft strength against a compressive load compared with a concentrically placed hole [32]. Frank correlated a greater drilling hole with higher torsional fracture risk [33]. Both may influence fracture risk even after implant healing. Ntalos et al. reported greater cortical destruction in 45° than in 90° inserted ASAs after anchor pullout [18]. Lacheta et al. pointed out that imbalanced loading of suture strands causes higher anchor displacement and decreased ultimate loads to failure [16], which is seen more often in 45° than in 90° inserted anchors. The latter can also explain the observed higher number of suture breakage in 90° than in 45° inserted 2.6 FiberTak® anchors (5/6 compared to 2/6) as the failure mode. Along with Barber and Herbert, we see significant differences in ultimate loads to failure and failure modes between anchor types [28], although we do not expect them to have clinical relevance.

With respect to the special insertion site of the bicipital groove, Otto, Lacheta and Chiang evaluated a range of ultimate loads to failure between 170 and 290 N for subpectoral LHB tenodesis with ASAs [9–11]. However, these studies were performed on human cadaveric bones with tendons attached. The major failure mode in these studies was tendon rupture first and suture breakage at the suture-tendon interface second. Therefore, all three tested anchors in our study can be regarded as suitable fixation methods for subpectoral biceps tenodesis, showing higher ultimate loads to failure overall. Tendon quality and suture parameters are possibly more important than the fixation method in determining ultimate failure loads. Interestingly, the standard deviation doubled in the 45° insertion angle in our study, and the lowest values in the 45° anchor series were 245 N for 1.6 FiberTak®, 197 N for 1.9 FiberTak® and 145 N for 2.6 FiberTak®. Hence, the anchor-bone interface might turn into the critical site of anchor failure when withstanding lower ultimate loads to failure than the suture-tendon interface. Therefore, we can only recommend that surgeons be aware of these facts and use a 90° insertion angle for secure ASA fixation.

### Limitations

This study still has limitations. First, the study was conducted on porcine humeri with higher bone density and cortical thickness; therefore, the results cannot be transferred to human humeri in all aspects. Second, the study protocol consisted of a quasi-static testing protocol. Cyclic loading and cyclic elongation were not assessed. Cyclic testing protocols may show different data. Finally, the study is a time-zero cadaveric study, and healing processes of the long biceps tendon to the bone as well as cyst formation cannot be depicted. Therefore, the external validity of the study is limited.

## Conclusion

All three suture anchors are suitable fixation methods for subpectoral biceps tenodesis. Regarding our data, we recommend 90° as the optimum insertion angle.

## Clinical relevance

All-suture anchors are a popular fixation method in biceps tenodesis. The influence of anchor size and insertion angle should be known by the surgeon for optimizing ultimate loads to failure and for achieving a secure fixation.

## Data Availability

The data sets used and/or analysed during the current study are available from the corresponding author.on reasonable request.

## Abbreviations

ASA All: Suture anchor
CSA: Conventional suture anchor
CT: Computer tomography
LHB: Long head of the biceps tendon
RC: Rotator cuff
